# Clearance of serum solutes by hemofiltration in dogs with severe heat stroke

**DOI:** 10.1186/s13049-014-0049-z

**Published:** 2014-08-22

**Authors:** Guang-Ming Chen, Yu-Ying Lan, Cheng-Feng Wang, Hai-Xia Zhan, Wen-Rui Wang, Jin-Hua Chen, Jian Chen

**Affiliations:** 1 Department of Pediatrics, Fuzhou General Hospital of Nanjing Military Command, PLA, Clinical Medical College of Fujian Medical University in Fuzhou General Hospital of Nanjing Military Command, PLA, Dongfang Hospital Affiliated to Xiamen University, NO. 156, Xi Er-Huan North Road, Fuzhou 350025, Fujian Province, China; 2 Fujian University of Traditional Chinese Medicine, Fuzhou 350108, China; 3 Department of Thoracic Surgery, Fuzhou General Hospital of Nanjing Military Command, PLA, Clinical Medical College of Fujian Medical University in Fuzhou General Hospital of Nanjing Military Command, PLA, Dongfang Hospital Affiliated to Xiamen University, Fuzhou 350025, China; 4 Statistics Room, Fuzhou General Hospital of Nanjing Military Command, PLA, Clinical Medical College of Fujian Medical University in Fuzhou General Hospital of Nanjing Military Command, PLA, Dongfang Hospital Affiliated to Xiamen University, Fuzhou 350025, China; 5 Department of Nephrology, Fuzhou General Hospital of Nanjing Military Command, PLA, Clinical Medical College of Fujian Medical University in Fuzhou General Hospital of Nanjing Military Command, PLA, Dongfang Hospital Affiliated to Xiamen University, NO. 156, Xi Er-Huan North Road, Fuzhou 350025, Fujian Province, China

**Keywords:** Clearance, Dogs, Heat stroke, Hemofiltration, Shock, Solute

## Abstract

**Background:**

We have previously reported that hemofiltration (HF) may be an effective additional means of treating heat stroke when rapid cooling is not effective.

**Methods:**

Dogs were assigned to a heat stroke (control) or heat stroke + hemofiltration (HF) group (n = 8 each group). After heat stroke induction, dogs in the HF group received HF for 3 h. Serum concentrations of interleukin (IL)-10, tumor necrosis factor (TNF)-α, IL-6, blood urea nitrogen (BUN) and creatinine were measured at baseline and 1, 2, and 3 h after heat stroke. Clearance rates of solutes were determined 1, 2, and 3 h after the start of HF.

**Results:**

Serum concentrations of all solutes tended to increase with time after heat stroke in the control group, but decreased (BUN, creatinine) or remained relatively unchanged (TNF-α, IL-6, IL-10) with time in the HF group. Concentrations of all solutes were significantly lower in the HF group compared with the control group at 2 and 3 h (*P* < 0.05). Clearance rates for small molecular weight solutes were high, while those for larger molecular weight solutes were low.

**Conclusion:**

HF prevents heat stroke-induced increases in serum cytokine concentrations and is effective for clearing small molecular weight solutes from serum, but less effective for clearing larger molecular weight solutes, including TNF-α, IL-6, and IL-10.

## Background

Heat stroke is a common condition indicated by an increased core body temperature (>40°C), central nervous system dysfunction, and often also cardiovascular dysfunction [1],[2]. Inflammatory and immunologic changes are also common [3],[4]. Of note, estimates suggest that there are approximately 600 heat-related deaths per year in the United States [5]. Furthermore, heat illness among high school athletes in the United States is the most common cause of morbidity and mortality [6]. The onset of heat stroke can be abrupt, and may result in multiple organ damage with consequent long term dysfunction or death [1],[7]. Prompt treatment is therefore essential to ameliorate hyperthermia and the risk of long term disability or death. The current standard of care for heat stroke is reducing core temperature by active cooling through cold water immersion [2],[8]. Unfortunately, such treatment is ineffective in approximately one-third of patients [9],[10]. As a consequence, we and others have been investigating additional methods for the treatment of heat stroke.

In a previous study, we reported that continuous hemofiltration (HF), a technique often used in the treatment of critically ill patients with acute renal failure in intensive care settings [11],[12], helped to rapidly reduce core body temperature, stabilize hemodynamics, improve homeostasis, and increase survival in dogs with severe heat stroke [13]. Zhou et al. have also recently reported that HF was effective for lowering core body temperature, removing myoglobin, improving organ function, and modulating acid–base balance and the systemic inflammatory response in 16 patients with heat stroke [14]. The precise mechanisms through which HF exerts these beneficial effects in heat stroke are unclear.

Because heat stroke is associated with a systemic inflammatory response [1], we hypothesized that the previously observed beneficial effects of HF in heat stroke [13] may at least in part be due to the removal of solutes from blood, especially inflammatory mediators. Indeed, various other studies have demonstrated that HF has beneficial effects that are presumably mediated by modulation of plasma/serum concentrations of inflammatory and anti-inflammatory mediators including tumor necrosis factor-α (TNF-α), interleukin-6 (IL-6), and interleukin-10 (IL-10) in animal models of endotoxin-induced lung injury [15], severe acute pancreatitis [16], and septic shock [17], and in clinical studies of patients with systemic inflammatory response syndrome/multiple organ dysfunction syndrome [18]. Therefore, in the present study, we investigated changes in serum concentrations and clearance of the pro-inflammatory factors TNF-α and IL-6 and the anti-inflammatory factor, IL-10, in dogs following the induction of heat stroke and HF. We also examined changes and clearance of two smaller molecular weight solutes, blood urea nitrogen (BUN) and creatinine.

## Methods

### Animals

Healthy male dogs (n = 16: purchased from the Department of Comparative Medicine, Fuzhou General Hospital of Nanjing Military Region) weighing 11–17 kg were housed in a temperature (24.7 ± 1.3°C) and humidity (49.9 ± 6.5%) controlled environment. Animals were randomly assigned to a control (heat stroke only) or HF (heat stroke + HF) group (n = 8 per group). The sample size used was selected with reference to previous studies [19]–[21], in which 6–8 animals were used per group. The study was approved by the Institutional Review Board of Fuzhou General Hospital of Nanjing Military Command.

### Anaesthesia

Animals were food-deprived for 6 h and then intramuscularly injected with ketamine hydrochloride (40 mg/kg) and atropine (0.05 mg/kg). During the study, anesthesia was maintained with low dose ketamine hydrochloride. After the induction of anesthesia, rectal lavage was performed with 200 mL of normal saline to remove residual faeces. A rectal probe (Philips Technology Co., Ltd, Beijing, China) was then inserted 10 cm toward the rectum for continuous monitoring of rectal temperature as the indicator of core temperature.

### Catheterization

After the induction of anesthesia, the skin above the femoral artery/vein was sterilized and an incision was made. The femoral artery and vein were exposed, and an 8-Fr double-lumen central catheter inserted in each. The arterial catheter was used for monitoring of arterial pressure and blood gases (Invasive Continuous Arterial Blood Pressure Monitor, Philips Technology Co., Ltd), and the venous catheter was used for HF and for collection of blood samples.

### Induction of heat stroke

Severe heat stroke was induced by exposing animals to a high temperature as previously described [13],[22]. Briefly, two dogs at a time (one from each group) were placed in a high temperature climate cabin (Nanfang Medical University, Guangdong, China) with a temperature of 36 ± 0.5°C and a humidity of 70% to simulate a megathermal climate. Severe heat stroke was indicated by a rectal temperature > 42°C and a transient decrease in mean arterial pressure > 25 mmHg. Upon induction of heat stroke, dogs were removed from the chamber, and those in the control group observed at room temperature, and those in the HF group subjected to HF.

### Hemofiltration

HF was carried out using a hollow fiber hemofilter with a polysulfone membrane (membrane area = 1.4 m^2^ with cut off value of 30 kDa; Fresenius, Oberursel, Germany). The circulation tube was first flushed with normal saline containing 25 IU/mL heparin for 30 min in order to heparinize the tube. Tube and filter were then flushed with 200 mL pre-collected blood (200 mL) in order to prevent anemia due to dilution. Next, the central catheters in the femoral artery and vein were connected to the arterial and venous ends of the machine, and hemofiltration was initiated. No ultrafiltration was done, so volume expansion did not occur. The only fluid added was only replacement of the fluid in the filtrate. Heparin (1250 IU/kg) was given via the femoral artery 1 min before HF, and maintenance heparin (625 IU/kg/h) was given during HF via a micro bolus pump. HF was performed for 3 h at a blood flow rate of 70 ml/min and a fluid replacement rate of 200 ml/kg/h. The replacement fluid contained 138.28 mmol/L Na, 106.58 mmol/L Cl, 35.72 mmol/L bicarbonate, 2.71 mmol/L Ca, 1.52 mmol/L Mg, 9.26 mmol/L glucose, and 4.02 mmol/L K. The temperature of the replacement fluid was 24.69 ± 1.28°C.

### Outcome measurements

Peripheral blood counts (white blood cells [WBC], neutrophils, leukocytes, red blood cells, and platelets) were the primary outcome measure and were determined before the induction of heat stroke (baseline), when heat stroke was first detected (0 h), and 1, 2, and 3 h after heat stroke.

Serum concentrations of 5 solutes, IL-10, TNF-α, IL-6, BUN, and creatinine, were also measured before the induction of heat stroke (baseline), when heat stroke was first detected (0 h), and 1, 2, and 3 h after heat stroke. Concentrations of TNF-α, IL-6, and IL-10 were measured using commercially available enzyme-linked immunosorbent assay kits (Beijing Meidike Biotechnology Co., Ltd., Beijing, China). Concentrations of BUN and creatinine were measured using an Olympus AU2700 automatic biochemical analyzer (Olympus Inc., Tokyo, Japan).

In the HF group, 2 mL of filtrate were collected at 1, 2, and 3 h, and centrifuged at 3000 r/min for 30 min. Ultrafiltrate concentrations of IL-10, TNF-α, IL-6, BUN, and creatinine were measured as already described.

Clearance rates for IL-10, TNF-α, IL-6, BUN, and creatinine were determined 1, 2, and 3 h after the start of HF [19]. Clearance rates were calculated as follow: Clearance = (E/P) × QE, where E and P are the concentrations of specific solutes in filtrate and serum, and QE is the liquid volume at the outflow end (=QD + QUF). QD refers to the amount of fluid displacement due to hemofiltration and QUF refers to the ultrafiltration-overflow volume. For this study no ultrafiltration was done, unlike the clinical studies that combine hemofiltration and ultrafiltation. Therefore, QUF was 0; and hence QE was equal to QD.

### Statistical analysis

**Figure 1:**
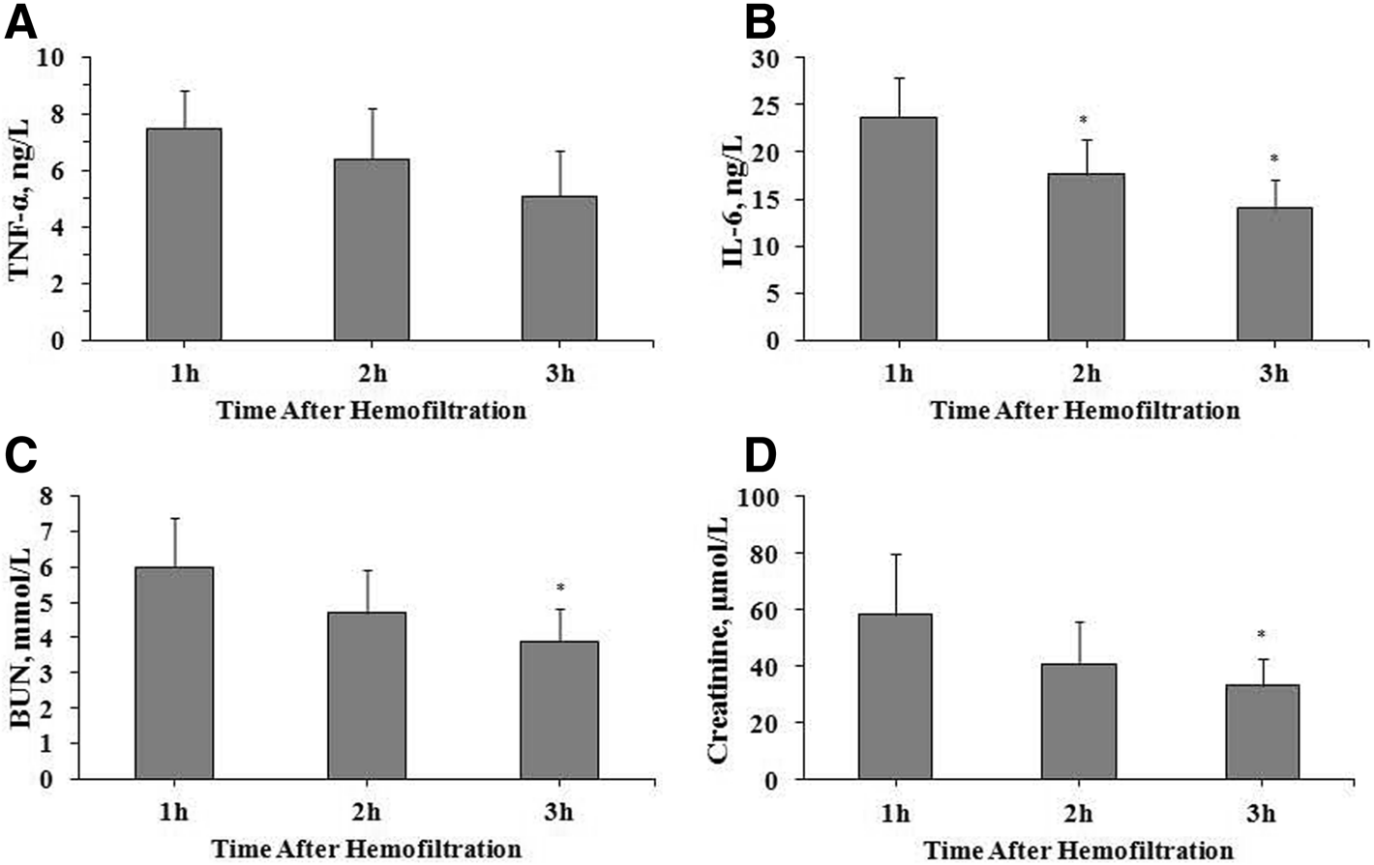
**Changes in concentrations in ultrafiltrate for (A) TNF-α, (B) IL-6, (C) BUN, and (D) creatinine after hemofiltration in dogs with heat stroke.** Data are presented as mean ± standard deviation and compared using ANOVA test with Bonferroni correction for post-hoc comparisons. *P < 0.0167 compared with the 1 h time point, after Bonferroni correction. IL-10 is not shown because it was not detected. Abbreviations: BUN, blood urea nitrogen; C, control; HF, hemofiltration; IL, interleukin; TNF, tumor necrosis factor.

**Figure 2:**
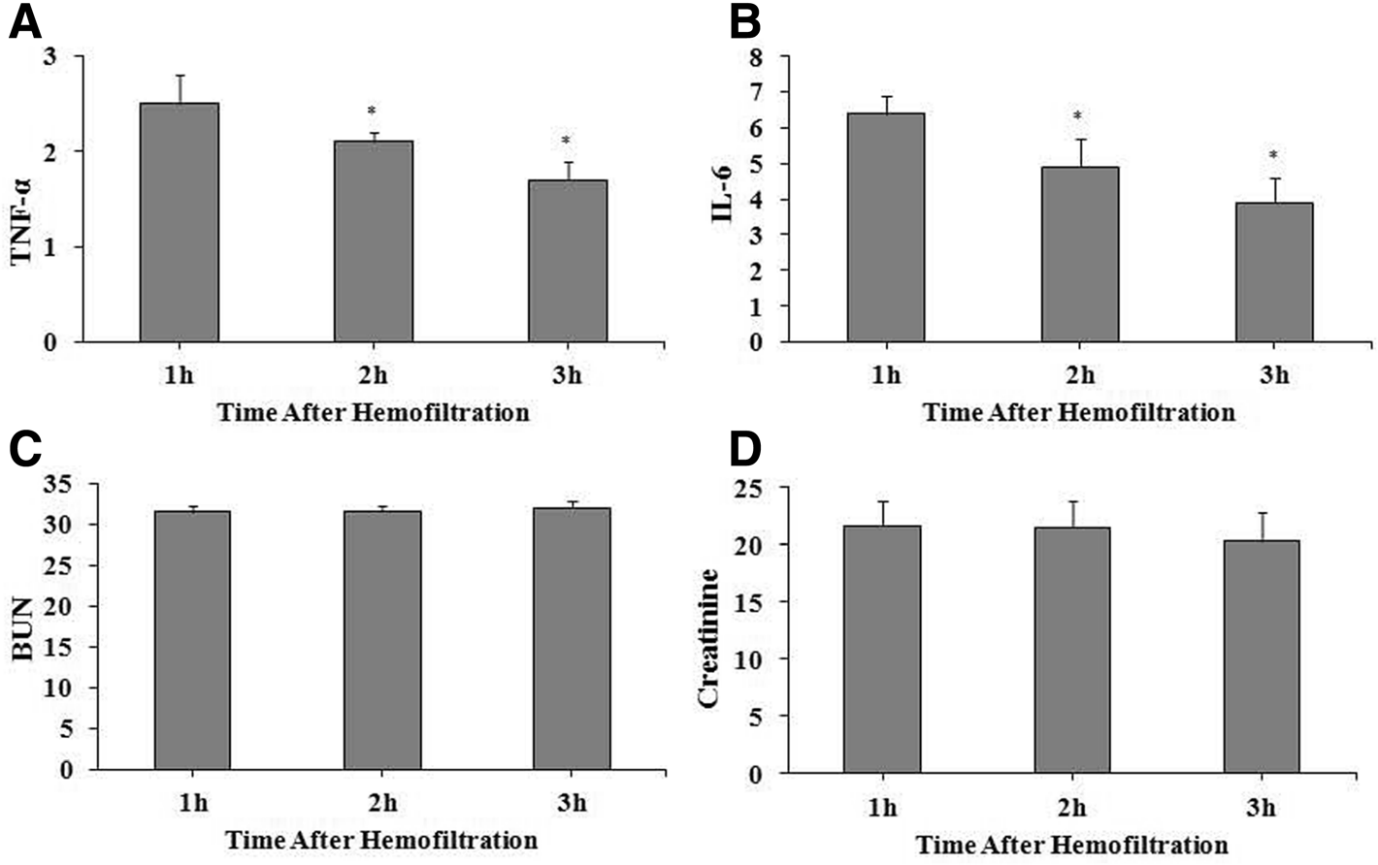
**Clearance rates after hemofiltration in dogs with heat stroke for (A) TNF-α, (B) IL-6, (C) BUN, and (D) creatinine.** Data are presented as mean ± standard deviation and compared using ANOVA test with Bonferroni correction for post-hoc comparisons. *P < 0.0167 (0.05/3) compared with the 1 h time point, after Bonferroni correction. IL-10 is not shown because it was not detected. Abbreviations: BUN, blood urea nitrogen; C, control; HF, hemofiltration; IL, interleukin; TNF, tumor necrosis factor.

Data are presented by mean ± standard deviation and were compared by analysis of variance with Bonferroni correction for post-hoc comparisons. Data were analyzed using SPSS 15.0 statistics software (SPSS Inc, Chicago, IL). The significance level was set at P < 0.05 for comparing the difference between control and HF groups at a given time point. An adjusted significance level of P < 0.01 (0.05/5) was used when comparing peripheral blood findings (Table 1) and solute concentrations (Table 2). And an adjusted significance level of P < 0.0167 (0.05/3) was used for comparing the change in concentration of solutes (Figure 1) and clearance rate for solutes after ultrafiltration (Figure 2). We could not use Cox regression model analysis for the data because we only measured outcomes at three time points after HF began, and therefore it was too hard to define the event and time-to-event parameters necessary for multiple outcome analysis.

**Table 1:** Comparison of peripheral blood findings between control and hemofiltration-treated dogs with heat stroke

**Cell**	**Group**	**Baseline**	**Heat stroke 0 h**	**Time after hemofiltration**		
				**1 h**	**2 h**	**3 h**
WBC, ×10^9^	C	10.52 ± 3.45	18.04 ± 5.6	20.72 ± 5.04	23.7 ± 5.19	23.41 ± 6.43
	HF	10.47 ± 3.46	19.63 ± 6.08	14.61 ± 4.44	13.03 ± 4.38	11.47 ± 3.73*
Neutrophil, %	C	74.99 ± 6.44	83.08 ± 5.16	83.29 ± 5.58	84.57 ± 7.8	85.25 ± 5.95
	HF	74.5 ± 8.24	82.49 ± 4.55	75.51 ± 4.96	75.44 ± 5.08	75.67 ± 4.79
Leukocyte, %	C	11.34 ± 4.42	7.16 ± 2.5	6.98 ± 2.58	5.87 ± 2.21	6.2 ± 1.65
	HF	11.36 ± 3.6	7.08 ± 1.5	8.13 ± 1.88	10.11 ± 2.66	10.68 ± 2.38
RBC, ×10^12^	C	5.92 ± 0.58	7.03 ± 1.07	7.09 ± 0.64	7.19 ± 1.01	7.2 ± 0.86
	HF	5.98 ± 0.61	7.13 ± 1.02	7.06 ± 0.63	7.08 ± 0.58	6.97 ± 0.7
PLT, ×10^12^	C	223.9 ± 54.5	149.4 ± 35.6	105.5 ± 18.4	78 ± 20.1	70.3 ± 22.3
	HF	236 ± 53.8	150.5 ± 37.1	117 ± 26.7	137.4 ± 41.4	138.3 ± 40.9

Data are presented as mean ± standard deviation.

C, control; HF, hemofiltration; PLT, platelet; RBC, red blood cell; WBC, white blood cell. *P < 0.05 significantly different between C and HF groups for given time points.

**Table 2:** Comparison of serum solute concentrations between control and hemofiltration-treated dogs with heat stroke

**Solute**	**Group**	**Baseline**	**Heat stroke 0 h**	**Time after hemofiltration**		
				**1 h**	**2 h**	**3 h**
TNF-α, ng/L	C	14.9 ± 2.6	93.6 ± 19.7	150.6 ± 20^†^	219.2 ± 31.7^†‡^	226.1 ± 27^†‡^
	HF	15.2 ± 2.6	94 ± 23.6	99.2 ± 19.6*	102.4 ± 22.7*	96.7 ± 19.9*
IL-6, ng/L	C	56.1 ± 7.4	128.5 ± 20.2	149.9 ± 13.4	167.4 ± 17.1	173 ± 15.5
	HF	56 ± 7.7	126.1 ± 15.6	122.6 ± 15.3	119.1 ± 17.3*	118.5 ± 19.2*
IL-10, ng/L	C	20.3 ± 2.8	65.8 ± 10.2	132.9 ± 13.6^†^	158.2 ± 12.5^†‡^	164.7 ± 12^†‡^
	HF	21 ± 3.6	63.2 ± 11.2	69.2 ± 8.4*	70.8 ± 8*	68.8 ± 7.7*
BUN, mmol/L	C	4.29 ± 0.82	10.5 ± 1.6	11.9 ± 1.3	12.6 ± 1.2	14.2 ± 1.3^†^
	HF	4.2 ± 0.9	10.6 ± 2.4	6.3 ± 1.4*^†^	5.0 ± 1.3*^†^	4.0 ± 0.9*^†^
Creatinine, μmol/L	C	59.9 ± 12.7	118.5 ± 21.3	156 ± 26.2	179.3 ± 22.5^†^	194.3 ± 21^†^
	HF	59.5 ± 12.2	122 ± 23.8	88.9 ± 29.5*	65.6 ± 20.4*^†^	54.9 ± 16.5*^†^

Data are presented as mean ± standard deviation. BUN, blood urea nitrogen; C, control; HF, hemofiltration; IL, interleukin; TNF, tumor necrosis factor.

*P < 0.05 significantly different between C and HF groups for given time points.

^†^*P* < 0.01 (0.05/5) compared with the 0 h time point in the same group, after Bonferroni correction.

^‡^*P* < 0.01 (0.05/5) compared with the 1 h time point in the same group, after Bonferroni correction.

## Results

### Baseline characteristics

Mean body weight was similar in the control and HF groups (14.03 ± 1.99 kg vs.14.09 ± 1.633 kg, *P* = 0.935). And consistent with our previous report [13], there were no between-group differences in other baseline characteristics, including body weight, temperature of the climate chamber, time to heat stroke, rectal temperature, ambient temperature, and ambient humidity (data not shown).

### Peripheral blood counts

Changes in peripheral blood counts for both groups are summarized in Table 1. The WBC count at 3 h was significantly lower in the HF group compared with the control group (*P* = 0.018). There were no other significant within or between-group differences in the peripheral blood count findings. The % neutrophil count was decreased and the % leukocyte and the platelet count increased in the HF group compared to the control group at all times during the HF, but none of these differences reached statistical significance.

### Serum solute concentrations

Changes in serum solute concentrations for both groups are summarized in Table 2. Heat stroke itself markedly increased the concentrations of all solutes. TNF-α, IL-10, BUN, and creatinine concentrations at 1, 2, and 3 h were significantly lower in the HF group compared with the control group. IL-6 concentrations at 2 and 3 h were significantly lower in the HF group compared with the control group, but at 1 h, IL6 concentrations showed only a trend to be lower in the HF group. When heat stroke was left untreated (control group), solute concentrations rose with time, and TNF-α and IL-10 concentrations were significantly higher at 1, 2, and 3 h, IL-6 and creatinine concentrations were significantly higher at 2 and 3 h, and BUN concentrations were significantly higher at 3 h compared to 0 h. In the HF group, BUN concentrations were significantly lower at 1, 2, and 3 h, and creatinine concentrations significantly lower at 2 and 3 h compared with at 0 h. And in the control group, TNF-α and IL-10 concentrations were significantly higher at 2 and 3 h compared to 1 h.

### Solute ultrafiltrate concentrations

The changes in solute ultrafiltrate concentrations (HF group only) are summarized in Figure 1. Except for IL-10, which was not detectable at any time point, concentrations of all solutes decreased with time after the start of HF. IL-6, BUN, and creatinine concentrations in ultrafiltrate were significantly lower at 3 h compared to 1 h (*P* < 0.001, *P* = 0.005, *P* = 0.015, respectively). IL-6 concentrations were also significantly lower at 2 h compared to 1 h (*P* = 0.008). There was no significant difference between any time points in TNF-α concentrations.

### Solute clearance rates

The changes in solute clearance rates (HF group only) are summarized in Figure 2. Clearance rates for small molecular weight solutes (BUN, creatinine) were higher than those for larger molecular weight solutes (TNF-α, IL-6). Clearance rates for IL-6 and TNF-α were significantly lower at 2 and 3 h compared to 1 h (2 h, *P* = 0.001 and 0.005 respectively; 3 h, both *P* < 0.001). There were no significant changes in the clearance rates for BUN or creatinine.

## Discussion

In this study, we examined serum concentrations and clearance of low and high molecular weight solutes (including cytokines) in dogs that received HF after the induction of heat stroke. Of note, during the 1 to 3 hour post-heat stroke induction period, serum concentrations of TNF-α, IL-6, IL-10, BUN, and creatinine were significantly lower in dogs that received HF than in dogs that did not receive HF. Clearance rates were relatively high for the low molecular weight solutes BUN and creatinine, but relatively low for the high molecular weight solutes TNF-α and IL-6. These findings suggest that (a) the previously observed biochemical beneficial effects of HF [13] may at least in part be due to the prevention of increases in serum concentrations of TNF-α, IL-6, and IL-10, and (b) that clearance of solutes by HF after heat stroke is dependent on molecular weight.

Hemofiltraion significantly decreased WBC compared to that seen in animals with no hemofiltration. Others have also reported hemofiltration to reduce WBC [23],[24]. It has been suggested that contact with the hemofilter causes leucocyte activation, an increase in leucocyte-platelet interaction and hemofilter clotting, and consequent decrease in WBC [25]. However, Skogby et al. [26] comparing fresh, heparinized blood perfused through an extracorporeal membrane oxygenation system for 24 h with or without addition of a hemofilter reported that addition of a hemofilter sis not significantly affect WBC or platelet count.

We found that in the 3 hours after the induction of heat stroke, serum concentrations of TNF-α, IL-6, IL-10, BUN, and creatinine continued to increase in the control group of dogs, but not in the group of dogs that subsequently received HF. Similarly, WBC counts increased in the control group after the induction of heat stroke, but had returned close to baseline after 3 h in the HF group. The increased cytokine concentrations and WBC counts observed in the control group are consistent with the systemic inflammatory response known to occur in heat stroke [1]. Because inflammation appears to play a role in mediating the deleterious effects of heat stroke [1],[27], the observed lack of further increase in cytokine concentrations in dogs that received HF is an important finding. This finding is also consistent with our previously reported finding that HF decreases serum concentrations of endotoxin, a key inflammatory mediator [1], in dogs with heat stroke [13]. Taken together, our findings suggest that the HF can ameliorate the systemic inflammatory response associated with heat stroke.

In the HF group, serum concentrations of BUN and creatinine decreased with time after the start of HF, whereas concentrations of TNF-α, IL-6, and IL-10 remained relatively steady. Consistent with these findings, the clearance rates were higher for BUN and creatinine than for TNF-α, and IL-6 (note: IL-10 was not detectable in ultrafiltrate samples). These findings suggest that HF is effective for clearing low molecular weight solutes, such as BUN and creatinine, but is less effective for clearing higher molecular weight solutes, such as cytokines. In general, hemofiltration has a lower clearance of low molecular weight molecules than for high molecular weight molecules. However, increasing the replacement rate in hemofiltration will increase he clearance of solutes, especially low molecular weight solutes [28], and the replacement flow rate used in our study (200 ml/kg.h) is a rate categorized by others as extremely high volume ultrafilratioon [29]. In our clinical practice, when extremely high hemofiltration was performed in patients with acute pancreatitis, similar findings were observed, that is, the concentration of small molecules such as urea and creatinine was reduced significantly. The low clearance rates for cytokines can be attributed to the molecular weight filtration capacity of the filter used (30 kDa). Note: the molecular weights for TNF-α, IL-6, and IL-10 are 19–28 kDa, 17.5 kDa, and 35–40 kDa, respectively. The lower molecular weights of TNF-α and IL-6 relative to IL-10 explains why these cytokines were cleared to some extent, whereas IL-10 was not cleared at all. A similar study has been reported elsewhere [30]. Use of a filter with a larger molecular weight filtration capacity or HF in combination with plasma replacement and hemoperfusion might help increase the clearance of larger molecular weight solutes [31],[32]. Further studies are needed to examine this possibility. Expansion of the blood volume had no role in the hemofiltration results, because the only fluid given in hemofiltration is fluid to replace the fluid removed in the filtrate.

Although serum cytokine concentrations did not decrease with HF, they did not increase after heat stroke as seen in the control group, and were significantly lower at these times than corresponding concentrations in control group dogs. We suggest that the lack of an increase in serum cytokine concentrations in the HF group may be at least in part due to the previously demonstrated early and rapid reduction in body temperature, improved homeostasis, and the lack of an increase in serum endotoxin concentrations following HF [13]. Increased clearance/removal of TNF-α and IL-6 through HF would also appear to play some role in preventing further increases in the serum concentrations of these cytokines. There also may have been some absorption of cytokines by the filter used for HF. Further studies are warranted to examine in more detail the relative contributions of filtration and changes in body temperature in mediating the potential beneficial effects of HF.

This study has a number of limitations. We did not measure soluble cytokine receptors and so we do not know how what role they might have played. We did not include a control group of normal (non-heat shock, non-HF) dogs, however the baseline blood factor values obtained from our dogs provided data on normal values for these factors. We did not include a control group of dogs that only received heat stroke, because the clearance of factors in blood by HF alone has previously been investigated by numerous animal and clinical studies. We did not include a group with active cooling because of cost concerns. Also, our objective was limited to determining the effect of HF after heat shock vs. heat shock alone. We did not include a group of animals treated with fluid infusion because none of the animals in our 2 groups received fluid infusion except for replacement of fluid lost during HF, and so fluid infusion was not involved. The total number of dogs included in each group (n = 8) was relatively small; hence, confirmatory studies are needed. We did not perform any assessments of kidney function (ie, urinary clearance of solutes). Finally, the duration of follow-up was relatively limited. Further studies are needed to examine longer term changes in serum solutes after HF, as well as short and longer term changes in urine solutes after HF.

## Conclusions

In summary, this study has shown that HF prevents increases in serum cytokine concentrations due to heat stroke in dogs, and that HF is effective for clearing small molecular weight serum solutes, but is less effective for clearing larger molecular weight solutes. Increased clearance of TNF-α and IL-6 by HF may play some role in preventing the increase in serum concentrations of these cytokines; however, the magnitude of clearance was not large relative to that of the smaller weight solutes. Taken together with our previously reported findings that HF rapidly reduces core body temperature, stabilizes hemodynamics, improves homeostasis, and increases survival compared to no treatment in dogs with severe heat stroke [13], these findings further emphasize the beneficial biochemical effects of HF in the treatment of heat stroke. Further studies are needed to elucidate the changes underlying these beneficial biochemical effects more fully and to optimize the use of HF as a potential treatment for heat stroke.

## Competing interests

The authors declare that they have no competing interests.

## Authors’ contributions

GMC: guarantor of integrity of the entire study; study design; definition of intellectual content; literature research; data analysis. YYL: study concepts; literature research; experimental studies; data acquisition. CFW: study concepts; clinical studies. HXZ and WRW: experimental studies. JHC: data acquisition; data analysis; statistical analysis. JC: guarantor of integrity of the entire study; study design; definition of intellectual content; manuscript review. All authors read and approved the final manuscript.
